# Protein Expression of Amino Acid Transporters Is Altered in Isolated Cerebral Microvessels of 5xFAD Mouse Model of Alzheimer’s Disease

**DOI:** 10.1007/s12035-022-03111-y

**Published:** 2022-11-11

**Authors:** Elena Puris, Liudmila Saveleva, Izaque de Sousa Maciel, Katja M. Kanninen, Seppo Auriola, Gert Fricker

**Affiliations:** 1 Institute of Pharmacy and Molecular Biotechnology, Ruprecht-Karls-University, Im Neuenheimer Feld 329, 69120 Heidelberg, Germany; 2 A.I. Virtanen Institute for Molecular Sciences, University of Eastern Finland, P.O. Box 1627, 70211 Kuopio, Finland; 3 School of Pharmacy, University of Eastern Finland, P.O. Box 1627, 70211 Kuopio, Finland

**Keywords:** Alzheimer’s disease, Membrane transporter, 5xFAD mice, Amino acids, Brain microvessels, Brain cortex

## Abstract

Membrane transporters such as ATP-binding cassette (ABC) and solute carrier (SLC) transporters expressed at the neurovascular unit (NVU) play an important role in drug delivery to the brain and have been demonstrated to be involved in Alzheimer’s disease (AD) pathogenesis. However, our knowledge of quantitative changes in transporter absolute protein expression and functionality in vivo in NVU in AD patients and animal models is limited. The study aim was to investigate alterations in protein expression of ABC and SLC transporters in the isolated brain microvessels and brain prefrontal cortices of a widely used model of familial AD, 5xFAD mice (8 months old), using a sensitive liquid chromatography tandem mass spectrometry-based quantitative targeted absolute proteomic approach. Moreover, we examined alterations in brain prefrontal cortical and plasmatic levels of transporter substrates in 5xFAD mice compared to age-matched wild-type (WT) controls. ASCT1 (encoded by Slc1a4) protein expression in the isolated brain microvessels and brain prefrontal cortices of 5xFAD mice was twice higher compared to WT controls (*p* = 0.01). Brain cortical levels of ASCT1 substrate, serine, were increased in 5xFAD mice compared to WT animals. LAT1 (encoded by Slc7a5) and 4F2hc (encoded by Slc3a2) protein expressions were significantly altered in the isolated brain microvessels of 5xFAD mice compared to WT controls (*p* = 0.008 and *p* = 0.05, respectively). Overall, the study provides important information, which is crucial for the optimal use of the 5xFAD mouse model in AD drug development and for investigating novel drug delivery approaches. In addition, the findings of the study shed light on the novel potential mechanisms underlying AD pathogenesis.

**Supplementary Information:**

The online version contains supplementary material available at 10.1007/s12035-022-03111-y.

## Introduction

Alzheimer’s disease (AD) is a prevalent neurodegenerative disease, which is the most common cause of dementia. AD is biologically defined by the presence of extracellular β-amyloid (Aβ) plaques and intraneuronal neurofibrillary tangles (NFTs) composed of aggregated hyperphosphorylated tau protein in the brain [1]. The amyloid plaques consist of insoluble aggregates of Aβ peptides, which are produced after the amyloid precursor protein (APP) cleavage by β- and γ-secretases. Moreover, there is growing evidence of dysfunction of the neurovascular unit (NVU) with the blood–brain barrier (BBB) in AD [2]. The BBB regulates the passage of solutes, such as nutrients and drugs, into and out of the brain by forming tight junctions between the brain microvascular endothelial cells and by expressing transporters as well as metabolizing enzymes [3].

The transporters expressed at the brain capillary endothelial cells and other cells of NVU including glial cells, such as astrocytes, microglia, as well as pericytes and neurons mainly refer to either Solute carrier family transporters (SLCs) or adenosine triphosphate (ATP)-binding cassette transporters (ABCs) [4]. ABC and SLC transporters of the NVU have been shown to play roles in AD pathogenesis by being involved in several molecular pathways altered in AD [5, 6]. It has been reported that extracellular Aβ accumulation in the brain is caused by reduced clearance of the peptides across the BBB due to the downregulation of ABCB1 transporter (also known as P-glycoprotein or multidrug resistance protein 1) [7, 8]. Moreover, the studies showed that, in addition to ABCB1, other ABC transporters regulate the clearance of Aβ from the brain [5, 6]. Importantly, several metabolic pathways, for example, amino acid and glucose homeostasis, have been found to be altered in AD brain, which could be caused by altered expression and/or function of SLC transporters regulating the influx of the nutrients and metabolites in the brain [6, 9–17]. Both ABC and SLC transporters play important roles in brain delivery of CNS drugs including donepezil, galantamine, rivastigmine, and memantine [18–21], which can be altered in AD resulting in unexpected therapeutic outcomes or adverse effects. Thus, the investigation of AD-related changes in transporter protein expression and function in the NVU cells is crucial for identifying new molecular mechanisms, which underly AD pathogenesis, development of effective AD treatments, investigating novel drug delivery strategies, and predicting drug concentrations in AD patients.

The majority of AD cases refer to late-onset AD which occurs after the age of 65 years, while the rarer form of AD, early-onset AD, accounts for less than 5% of all cases and manifests by age 60 [22]. Most patients who suffer from early-onset AD have familial AD (FAD), which is a hereditary disease associated with mutations in the genes involved in the production of Aβ peptides such as APP and presenilin-1 and 2 (PSEN1 and PSEN2) [23]. Although FAD is much less common form of AD compared to sporadic AD, the mutations associated with this AD form underlie the major molecular representation of AD based on which many of the animal models used in AD drug development have been designed, as well as drug candidates have been developed and tested. One of the FAD models widely used in AD research is a transgenic 5xFAD mouse, which expresses human APP and PSEN1 transgenes with five mutations linked to AD [24]. The 5xFAD model is characterized by amyloid plaque pathology, which is more pronounced in females than in males, cognitive impairment, and inflammation similar to that found in AD patients with an accelerated rate compared to other models of FAD [24–27]. However, information about changes in the NVU transporter protein expression and function in this mouse AD model is limited. Therefore, quantitative information about the expression of NVU transporters in 5xFAD mice is crucial for understanding the relevance of the use of the model to mimic AD pathology during the investigation of the molecular mechanisms, which underly the progression of AD, drug target validation, development of new brain-targeting drug delivery strategies, and translation of preclinical data to humans.

The aim of the present study was to characterize a mouse model of familial AD, 5xFAD mice [24], which is widely used in AD research and drug development, in terms of changes in protein expression of five ABC and thirteen SLC transporters in the isolated brain microvessels as well as the brain prefrontal cortex. Here, we applied previously developed sensitive liquid chromatography with tandem mass spectrometry (LC–MS/MS)-based quantitative targeted absolute proteomics (QTAP) methods [28, 29]. The LC–MS/MS-based QTAP approach enables a sensitive and robust quantitative analysis of low abundant target proteins with multiple advantages over the antibody-based methods such as western blotting [30], and has been extensively applied for protein expression quantification of transporters and other proteins in the NVU in health and diseases [17, 28, 29, 31–35]. Moreover, we compared the alterations in transporter protein expression in the isolated brain microvessels and brain prefrontal cortices of 5xFAD mice to previously published data in AD patients and other animal models in order to assess the relevance of the animal models to mimic AD-related changes in transporter protein expression in the NVU in humans. Finally, we investigated if there are any changes in the brain prefrontal cortical and plasmatic concentrations of the substrates of the transporters, whose expression have been found to be altered in 5xFAD mice, in order to provide indirect evidence of the contribution of these transporters to AD pathogenesis in the functional level.

## Materials and Methods

### Materials

Dithiothreitol (#D9760), guanidine hydrochloride (#G3272), acetonitrile (#1.00029), formic acid (5.33002), ethylenediaminetetraacetic acid (EDTA) (#E9884), Tris–HCl (#10,812,846,001), bovine serum albumin (BSA, A3294), NaCl (#S9888), KH_2_PO_4_ (#P5655), KCl (#P3911), HEPES (#H3375), CaCl_2_·2H_2_O (#223,506), MgSO_4_·7H_2_O (#1,374,361), dextran (#31,390), non-labelled amino acids L-serine (#PHR1103), L-alanine (#PHR1110), L-leucine (#PHR1105), L-isoleucine (#PHR1099), L-tyrosine (#PHR1097), L-phenylalanine (#PHR1100), histidine (#H0750000), and protease inhibitor cocktail (#11,836,170,001) were purchased from Sigma-Aldrich (St. Louis, MO). The stable isotope-labelled peptides (absolutely quantified) were provided by the JPT Peptide Technologies GmbH, Berlin, Germany. ProteoExtract® Subcellular Proteome Extraction Kit (#539,790) was purchased from Merck KGaA, Darmstadt, Germany. Tosylphenylalanylchloromethyl ketone-treated trypsin (VA9000), Protease-Max surfactant (V2072), and lysyl endopeptidase (Lys-C, VA1170) were purchased from Promega (Madison, WI, USA). A mixture of standards (product number MSK-A2-1.2) containing stable isotope-labelled amino acids was obtained from Cambridge Isotope Laboratories, Inc.

### Study Design and Experimental Model

The study was performed according to the Council of Europe Legislation and Regulation for Animal Protection. The approval for all the experiments (license number ESAVI-2018–012,856) was obtained from the Animal Experiment Board in Finland (Regional State Administrative Agency of Southern Finland). The animal experiments complied with the ARRIVE guidelines and were conducted according to EU Directive 2010/63/EU for animal experiments. In the present study, we used transgenic hemizygous 5xFAD mice (25 ± 2.7 g, *n* = 35) (RRID:MMRRC_034848-JAX, Jackson Laboratories, Bar Harbor, ME, USA) carrying human APP with the APP Swedish, Florida, and London mutations as well as human PSEN1 with the M146L and L286V mutations, which were driven by the mouse Thy1 promoter [36] and their wild-type (WT) littermates (20 ± 1.7 g, *n *= 35) on the C57BL/6 J background (RRID:IMSR_JAX:000,664, Jackson Laboratories, Bar Harbor, ME, USA). All mice were 8-month-old female mice as at this age 5xFAD female mice have been shown to develop a full range of AD pathological characteristics, such as amyloid pathology, presence of phosphorylated tau aggregates in the brain, cognitive impairment, long-term potentiation and synaptic transmission deficits, and astrocyte reactivity [24–27]. Due to a higher prevalence and risk of AD for women compared to men [37], female mice were used in the study. Mice were housed in controlled temperature and humidity, 12:12-h light–dark cycles, with access to water and a maintenance diet ad libitum. The animals were housed in plastic cages, which were covered by a reusable filter animal cage cover from Tecniplast Inc., USA, on aspen wood chips in Scantainer-units. This is an exploratory study, but to orientate in sample size, we used Abcg2 protein expression levels at the isolated rat brain microvessels from our previous study [28]. The sample size calculation for the two-sided *t*-test with 5% significant level and 80% power resulted in 6 observations per group.

### Mouse Genotyping

Transgenic 5XFAD mice (RRID:MMRRC_034848-JAX) and C57BL/6 J mice (RRID:IMSR_JAX:000,664) were purchased from The Jackson Laboratories (Jackson Laboratories, ME, USA). For maintaining the 5xFAD and non-transgenic WT colonies on site, male transgenic hemizygous 5xFAD mice were crossed with female C57BL/6 J dams at sexual maturity age (approximately 6 weeks of age). Due to the congenic background of 5xFAD mice, non-transgenic littermates (WT) were used as a control in the study. After pups were weaned, they were ear-punched for subsequent identification and genotyping. Mice were housed together with their littermates (*n* = 3–4 per cage) and aged until the study age.

For genotyping, DNA was isolated from mouse ear biopsies using 50 mM NaOH (Fisher Chemicals, #1,870,577) at 95 °C for 1 h followed by vortexing. Samples were then cooled to room temperature and 1 M Tris, pH 8.0 (Sigma, #T1503), was added to neutralize the lysis. Samples were centrifugated for 6 min at 13000 g at room temperature. The standard polymerase chain reaction (PCR) assay (Genotyping protocol database, #003,378, standard PCR Assay – Tg (PSEN1), Jackson Laboratories, Bar Harbor, ME, USA) was performed with obtained DNA samples and primers for the PSEN1 transgene (IDT, USA). All the primers are listed in Table S1. After PCR, samples were separated by gel electrophoresis on a 1% agarose gel (Nippon Genetics Europe, #AG02) with Tris/Borate/EDTA (TBE) buffer and pre-stained with Midori Green Advance DNA stain (Nippon Genetics Europe #MG04) for easier detection. Bands were detected with BioRad ChemiDoc™ MP Imaging system using ImageLab software (Bio-Rad, USA). Transgenic samples were identified by the presence of double bands of the PSEN1 transgene (608 bp) and internal positive control (324 bp). The mice lacking the PSEN1 transgene band were considered WT. A representative image of genotyping samples is provided in Supplementary File 1 (Fig. 4).

### Tissue Collection

On the day of tissue collection, at light time, mice were killed using carbon dioxide asphyxiation, as it has been used in previous studies to which the results of the present study are compared. In addition, the use of anaesthetics might affect the expression of transporters. Transcardial perfusion using heparinized 0.9% saline (2500 IU/L, LEO) was performed to remove the blood. After that, mouse brains were dissected out of the skull, excised, and the cerebrums were extracted. For the immediate microvessel isolation, the brains were placed on ice cold buffer A (pH 7.4) consisting of 101 mM NaCl, 1.2 mM KH_2_PO_4_, 4.6 mM KCl, 15 mM HEPES, 5 mM CaCl_2_·2H_2_O, and 1.2 mM MgSO_4_·7H_2_O. Prefrontal cortex of about 15 mg was snap frozen, and stored at −70 °C until the quantitative real-time polymerase chain reaction (qRT-PCR) experiment, Aβ ELISA analysis, and LC–MS/MS-based proteomic analysis and amino acid analyses.

### Aβ ELISA Assays

Total concentrations of Aβ_1–40_ and Aβ_1–42_ were measured in mouse brain cortical tissues as described previously [28] using Human Aβ_40_ and Aβ_42_ ELISA Kits purchased from Thermo Fisher Scientific (KHB3481 for Aβ_40_ and KHB3544 for Aβ_42_), following the manufacturer’s instructions. The concentrations of Aβ_1–40_ and Aβ_1–42_ were normalised to total protein levels (as presented by micrograms of Aβ per gram of total protein), which were measured using Bio-Rad DC Protein Assay (#5,000,112).

### qRT-PCR Analysis

Expression of inflammation markers, such as a marker of microglial activation, allograft inflammatory factor 1 (Aif1), and a marker of abnormal activation and proliferation of astrocytes, glial fibrillary acidic protein (Gfap), as well as a pro-inflammatory cytokine, interleukin-1 beta (Il1b), were quantified by qRT-PCR analysis in brain cortices of WT and 5xFAD mice. For this purpose, RNeasy Mini Kit (#74,004), Qiagen, Stockach, Germany, was used for total RNA extraction from mouse brain cortices according to the manufacturer’s protocol. Total RNA was quantified by NanoDrop, Thermo Scientific, Dreieich, Germany. After that, cDNA was synthetized with Biozym cDNA synthesis Kit (#331475S), Oldendorf, Germany, according to the manufacturer’s protocol. The obtained cDNA was mixed with the PowerUp ™ SYBR ™ Green Master Mix (#A25741) purchased from Thermo-Fischer, Waltham, USA, and different gene-specific primers (Table S2), which were obtained from Thermo Fisher Scientific. Relative target gene expression normalized to housekeeping gene expression, i.e., glyceraldehyde‐3‐phosphate dehydrogenase (Gapdh), in each sample was estimated according to the method explained previously [38]. For qRT-PCR analysis, LightCycler 96 (Roche Diagnostics) was used, and the data were acquired with LightCycler® 96 SW 1.1 software, v. 1.1.0.1320 (Roche Diagnostics, Mannheim, Germany; 2011).

### Isolation of Mouse Cerebral Microvessels

Mouse cerebral microvessels were isolated using the method combining a dextran density gradient separation with size filtration in accordance with the previously validated protocol [28], which has been regularly applied for transporter protein quantification studies in humans and animals with LC–MS/MS-based QTAP analyses [17, 39]. The cerebral microvessel isolation procedure was performed at 4 °C. Pooled mouse brain cortices (3.0 ± 0.2 g, 6–7 brain cortices per one brain microvessel sample) were dissected into 1 mm pieces. Five volumes of Buffer A per gram of tissue weight were added, and the cortices were homogenised by the Potter–Elvehjem homogenizer (DWK Life Sciences, Wheaton, 358,044) with 20 up-and-down, unrotated strokes. The obtained homogenates were centrifuged for 10 min at 2000 × *g* at 4 °C. The pellet was suspended in Buffer B, which consisted of Buffer A with 16% dextran, followed by centrifugation for 15 min at 4500 × *g* at 4 °C. After that, the supernatant was removed to a new tube, and centrifuged again for 15 min at 4500 × *g* at 4 °C. The pellets obtained after two centrifugation steps were suspended in Buffer C consisting of Buffer A with 5 g/L BSA and combined, followed by passing through a pre-wet nylon mesh of 200 µm (PluriStrainer® 200 µm, #43–50,200-03, PluriSelect Life Science, Germany). Consequently, the mesh was washed with 10 mL of Buffer C. The suspension, which passed through the filter, was then loaded onto a pre-wet nylon mesh of 100 µm (PluriStrainer® 100 µm, #43–57,100-51, PluriSelect Life Science, Germany). The nylon mesh was washed with 10 mL of Buffer C, and the obtained suspension was transferred to a pre-wet nylon mesh of 20 µm (PluriStrainer® 20 µm, #43–50,020-03, PluriSelect Life Science, Germany). The 20 µm mesh was washed with 40 mL of Buffer C, and the cerebral microvessels retained on the mesh were immediately collected after diverting the nylon mesh and washing it with 30 mL of Buffer C. The resulting suspension of the isolated cerebral microvessels was centrifuged for 5 min at 1000 × *g* at 4 °C. The obtained pellet consisting of the isolated brain microvessels was suspended in 1 mL of Buffer A. Consequently, the microscopic evaluation of the purity of the brain microvessels was performed. Finally, the suspension was centrifuged for 5 at 1000 × *g* min at 4 °C, and the supernatant was completely removed, while the resulting pellet was processed for crude membrane isolation using ProteoExtract® Subcellular Proteome Extraction Kit (#539,790) according to the manufacturer’s protocol. Subsequently, total protein levels were measured in the crude membrane fractions of the isolated cerebral microvessel samples using the Bio-Rad DC Protein Assay. The crude membrane fractions were stored at −80 °C until LC–MS/MS-based QTAP analysis.

In addition to microscopic examination, the purity of the isolated cerebral microvessel preparations was assessed by comparing the absolute protein expression of the plasma abluminal membrane marker, such as Na^+^/K^+^-ATPase, and the endothelial luminal membrane marker γ-glutamyltransferase (γ-Gtp), between the crude membrane fractions of isolated cerebral microvessels and corresponding mouse brain cortical tissue [40].

### Quantitative Targeted Absolute Proteomic Analysis

In the present study, the absolute protein expression of five ABC and thirteen SLC transporters (Table S3), Na^+^/K^+^-ATPase, and γ-Gtp was quantified in crude membrane fraction of the isolated cerebral microvessels and brain prefrontal cortices in WT and 5xFAD mice. The investigated transporters have been selected previously [28] based on the potential or proven contribution to AD [5, 6, 9–17]. For LC–MS/MS-based QTAP analysis, the samples were prepared according to the previously published protocol [41]. Briefly, for solubilization, 7 M guanidine hydrochloride, 500 mM Tris–HCl (pH 8.5), and 10 mM EDTA were added to the aliquots of samples (50 µg of total protein). Subsequently, the reduction of total protein was performed by addition of dithiothreitol, while *S*-carbamoylmethylation was performed by adding iodoacetamide. The precipitation with methanol and chloroform was performed on the samples. The resulting precipitates were dissolved in 6 M urea in 0.1 M Tris–HCl (pH 8.5). The samples were diluted 5 times by adding 0.1 M Tris–HCl (pH 8.5) containing internal standard peptides (Table S3). After this step, Lys-C and Protease-Max were added, and the samples were incubated for 3 h at room temperature. Finally, a 16-h tryptic digestion of the samples was performed by adding tosylphenylalanyl chloromethyl ketone-treated trypsin (enzyme to substrate ratio of 1:100) at 37 °C. The samples were acidified by addition of formic acid in water 20% (v/v) and centrifuged for 5 min at 14,000 × g at 4 °C. The resulting supernatants were used for LC–MS/MS-based QTAP analysis.

The LC–MS/MS analysis was performed with an Agilent 6495 Triple Quadrupole Mass Spectrometer equipped with an ESI source (Agilent Technologies, Palo Alto, CA, USA) coupled to an Agilent 1290 Infinity LC (Agilent Technologies, Waldbronn, Germany) system. The Advance Bio Peptide Map column (2.1 × 250 mm; 2.7 μm) was used for HPLC separation and elution of the peptides as reported previously [28]. Simultaneous detection was performed for the eluted peptides by applying the positive ion multiple reaction monitoring (MRM) mode with a dwell time of 20 ms per MRM transition. The following parameters were used: source temperature of 210 °C, drying gas flow rate of 16 L/min, nebulizer pressure of 45 psi, and MS capillary voltage of 3 kV. The data acquisition was performed with the Agilent MassHunter Workstation Acquisition software, Agilent Technologies, Data Acquisition for Triple Quad., version B.03.01. The data was processed with Skyline software (version 4.1).

The target protein expression quantitation was performed using one unique peptide (Table S3), which has been selected based on the peptide selection criteria in silico [41] and previous studies [28, 39, 42–44]. The respective MRM transitions (three to four) for each individual peptide linked to the fragment ions with high intensity were used for quantification of a stable isotope-labelled peptide and an unlabelled target peptide (Table S3). The average of three or four quantitative values was used for the calculation of protein expression. When only two or one transition(s) were obtained for the peptide, the expression of the corresponding protein was considered to be under the limit of quantification (ULQ). The quantification limits for each target protein were obtained as the lowest concentration of a stable isotope-labelled peptide producing signal peaks with three or four transitions. The protein expression in crude membrane fractions of the isolated mouse cerebral microvessels and brain prefrontal cortex was presented as absolute values (fmol/μg total protein).

### Amino Acid Analysis

#### Preparation of Plasma Samples

Mouse plasma samples were prepared in accordance with previously reported protocols with minor modifications [45, 46]. Briefly, plasma samples (30 μL) were centrifuged for 10 min at 14,000 × g at 4 °C. The supernatants (20 μL) were mixed with pre-chilled methanol (80 μL) containing internal standards (stable isotope-labelled amino acid mixture) to produce a final 80% (v/v) methanol solution. Subsequently, the samples were gently shaken and incubated for 6 h at −80 °C, followed by centrifugation for 10 min at 14,000 × g at 4 °C. The resulting supernatant (80 μL) was immediately used for the LC–MS/MS analysis described below.

#### Brain Cortical Tissue Sample Preparation

The brain prefrontal cortical tissue samples were prepared in accordance with the previously reported protocol with small modifications [43]. Briefly, 10–20 mg of brain cortical tissues were homogenised in 80% methanol in water (1:20, w/v) containing amino acid internal standards (stable isotope-labelled amino acid mixture). The samples were centrifuged for 10 min at 14,000 × g at 4 °C. Then, 70 μL of supernatant was mixed with 70 μL of 80% methanol in water containing internal standards and immediately used for the LC–MS/MS analysis described below.

#### Relative Quantification of Amino Acids in Mouse Plasma and Brain Prefrontal Cortical Tissue

For relative quantification of serine, alanine, phenylalanine, tyrosine, leucine, isoleucine, and histidine, an Agilent 1200 Series Rapid Resolution LC System (Agilent Technologies, Waldbronn, Germany) coupled with Agilent 6410 Triple Quadrupole Mass Spectrometer with an electrospray ionization source ESI (Agilent Technologies, Palo Alto, CA, USA) was used. The separation was performed using hydrophilic interaction chromatography (HILIC) in positive mode ESI in accordance with the previously described method [43, 47] using an ACQUITY UPLC BEH Amide column (100 mm × 2.1 mm, 1.7 μm; Waters Corporation, Milford, MA, USA). A flow rate was 0.6 mL/min. Column oven temperature was 45 °C. Eluent A was 50% (v/v) acetonitrile in water, while eluent B was 90% (v/v) acetonitrile in water, both containing 20 mM ammonium formate (pH 3). The gradient elution was as follows: (0–2.5 min) 100% B; (2.5–10 min) 100% B → 0% B; (10–10.01 min) 0% B → 100% B; and (10.01–12.5 min) 100% B. The injection volume was 2 μL. Mass spectrometric detection was performed as described previously [48] with MRM in the positive mode with the dwell time of 50 ms per transition and fragmentor voltage of 380 V. The MRM transitions, collision energies, and accelerator voltages are presented in Table S4. Data was acquired using the software Agilent MassHunter Workstation Acquisition (Agilent Technologies, Data Acquisition for Triple Quad., B.03.01) and processed with Quantitative Analysis (B.04.00) software. The methods were determined to possess acceptable (≤ 15% of the relative standard deviation) precision and accuracy.

### Statistical Analysis

The absolute protein expression levels are expressed as mean ± standard deviation (SD) as well as a ratio of protein expression between WT and 5xFAD. The total concentrations of Aβ_1–40_ and Aβ_1–42_, normalized fold expression of Gfap, Aif1, and Il1b, and amino acid levels in plasma and brain cortical tissue of 5xFAD mice are presented as percentage of control in WT mice (mean ± SD). Statistical significance of differences in absolute protein expression, amino acid levels, total concentrations of Aβ_1–40_ and Aβ_1–42_, and normalized fold gene expression between WT and 5xFAD mice was analyzed using an unpaired *t*-test. A *p*-value of less than 0.05 was considered to be statistically significant. A ROUT test was used to identify statistical outliers which were removed from the analysis. To confirm the normal distribution, the data were tested against the null hypothesis. Data analysis was done using GraphPad Prism, version 5.03, GraphPad Software, San Diego, CA.

## Results

### Characterization of the 5xFAD Mouse Model

**Fig. 1:**
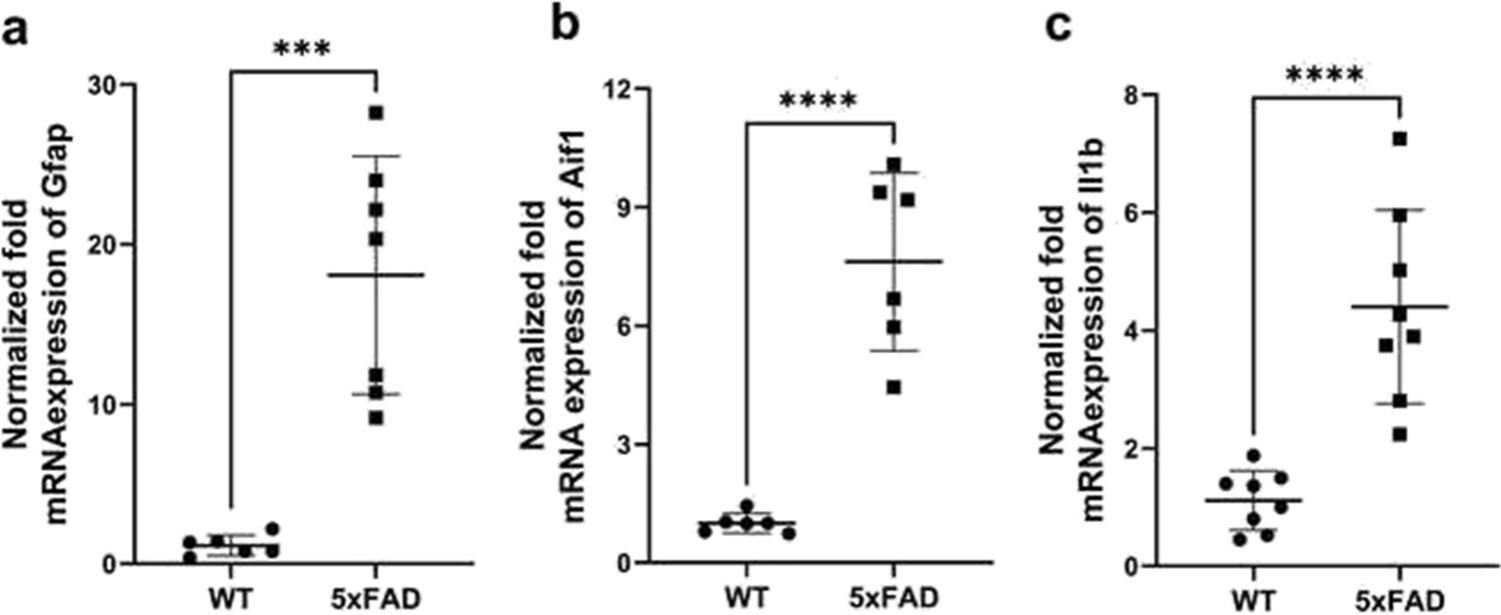
Normalized fold mRNA expression of inflammation markers, such as **a** glial fibrillary acidic protein (Gfap); **b** allograft inflammatory factor 1 (Aif1); **c** interleukin-1 beta (Il1b) in the brain prefrontal cortices of 5xFAD mice (*n *= 7) and wild-type (WT) control mice (*n *= 6). The normalization of gene expression was performed against the housekeeping gene glyceraldehyde‐3‐phosphate dehydrogenase (Gapdh). The data are expressed as mean ± SD. Asterisks denote a statistically significant difference from the respective control (***p < 0.005, **** < 0.001, unpaired *t*-test)

Female 5xFAD mice, as a model of familial AD, have been previously characterized in terms of AD pathology [25–27]. In this study, we confirmed that 5xFAD mouse model is characterized by Aβ pathology and inflammation in the brain. Thus, total Aβ_1–40_ and Aβ_1–42_ concentrations in the brain cortices of 5xFAD mice were 1.0 ± 0.67 mg/g total protein and 0.24 ± 0.098 mg/g total protein, respectively, while in WT mouse cortical tissue, the Aβ_1–40_ and Aβ_1–42_ were not detected (ULQ < 1.56 pg/mL for Aβ_1–42_ and ULQ < 7.81 pg/mL for Aβ_1–40_). In addition, the mRNA expression of inflammation markers, such as a marker of abnormal activation and proliferation of astrocytes, Gfap, a marker of microglial activation, Aif1, and pro-inflammatory cytokine interleukin-1 beta (Il1b), was significantly increased in the brain cortex of 5xFAD mice compared to WT controls (Fig. 1a–c).

### Transporter Protein Expression in the Isolated Cerebral Microvessels and Brain Cortices of 5xFAD Mice

In the present study, absolute protein expressions of five ABC and thirteen SLC transporters were quantified in crude membrane fractions of the isolated cerebral microvessels of WT and 5xFAD mice. The purity and enrichment of the isolated cerebral microvessels were confirmed microscopically (Supplementary File 1, Fig. 5). The protein expression of endothelial cell marker γ-Gtp in the crude membrane fractions of isolated brain microvessels in WT and 5xFAD mice was similar (Table 1), whereas in the corresponding brain prefrontal cortical tissues, the protein expression was under limit of quantification (ULQ < 0.03 fmol/μg total protein), indicating about comparable enrichment of the brain capillary endothelial cells over the brain prefrontal cortical tissue. Moreover, protein levels of the plasma membrane marker Na^+^/K^+^-ATPase in the crude membrane fractions of the isolated cerebral microvessels in both study groups did not differ (Table 1), demonstrating comparable enrichment of obtained membrane fraction between the samples in both study groups. Thus, the observed differences in transporter protein expression reported below were not caused by the inter-sample differences in the purity of the extracted membrane fractions.

**Table 1:** Absolute protein expression of ABC and SLC transporters (fmol/μg crude membrane total protein) in crude membrane fraction of the isolated brain microvessels in wild-type (WT) mice (*n* = 6) and 5xFAD mice (*n* = 6), as well as comparison to protein expression levels in crude membrane fraction of the isolated brain microvessels in WT rats (*n* = 6) and TgF344-AD rats (*n* = 6) [28] and in plasma membrane of the isolated brain microvessels in AD patients (*n* = 5) versus non-demented (ND) individuals (*n* = 12) [17]

Protein name/ gene name	Isolated mouse brain microvessels in the present study				Isolated rat brain microvessels [28]						Isolated human brain microvessels [17]	
	WT mice Mean ± SD	5xFAD mice Mean ± SD	Ratio 5xFAD to WT	*p*-value	WT rats Mean ± SD	TgF344-AD rats Mean ± SD	Ratio TgF344-AD to WT		*p*-value		ND individuals Mean ± SD	AD patients Mean ± SD
ABC transporters												
Abcb1/Abcb1^a^	3.1 ± 1.7	2.2 ± 1.0	0.71	0.3	1.6 ± 0.52	2.2 ± 0.61	1.3		0.1		2.6 ± 0.93	2.3 ± 1.5
Abcg2/Abcg2	1.1 ± 0.38	1.1 ± 0.44	1.0	0.9	0.12 ± 0.040	0.37 ± 0.18	3.1		0.01		2.2 ± 0.61	1.9 ± 0.84
Abcc1/Abcc1	0.065 ± 0.028	0.068 ± 0.037	1.1	0.8	0.021 ± 0.007	0.041 ± 0.004	2.0		< 0.01		< 0.050 (ULQ)	< 0.060 (ULQ)
Abcc4/Abcc4	0.13 ± 0.039	0.16 ± 0.062	1.2	0.3	0.046 ± 0.025	0.052 ± 0.034	1.1		0.7		NQ	NQ
Abca1/Abca1	0.092 ± 0.052	0.097 ± 0.041	1.1	0.8	0.012 ± 0.009^a^	0.033 ± 0.022	2.8		0.09		NQ	NQ
SLC transporters												
ASCT1/Slc1a4	0.71 ± 0.48	1.5 ± 0.36	2.1	0.01	0.17 ± 0.10	0.20 ± 0.18	1.2		0.7		NQ	NQ
GLUT1/Slc2a1	46.0 ± 22.0	49.0 ± 21.0	1.1	0.8	19.0 ± 7.6	20.0 ± 6.5	1.1		0.7		22.0 ± 9.8	18.0 ± 13.0
4F2hc/Slc3a2	0.50 ± 0.16	0.72 ± 0.17	1.4	0.05	0.43 ± 0.22	0.42 ± 0.24	1.0		0.9		NQ	NQ
CAT-1/Slc7a1	< 0.15 (ULQ)	< 0.15 (ULQ)			0.49 ± 0.21	0.53 ± 0.12	1.1		0.7		NQ	NQ
LAT1/Slc7a5	0.32 ± 0.14	0.12 ± 0.05	0.38	0.008	< 0.020 (ULQ)	< 0.020 (ULQ)					0.59 ± 0.15	0.56 ± 0.14
MCT1/Slc16a1	0.11 ± 0.07	0.097 ± 0.047	0.88	0.7	< 0.10 (ULQ)	< 0.10 (ULQ)					5.4 ± 3.7	3.1 ± 1.3
RFC/ Slc19a1	0.088 ± 0.043	0.099 ± 0.033	1.1	0.6	< 0.15 (ULQ)	< 0.15 (ULQ)					NQ	NQ
OATP1A4/ Slco1a4^b^	0.36 ± 0.19	0.49 ± 0.16	1.3	0.2	< 0.015 (ULQ)	< 0.015 (ULQ)					0.54 ± 0.10	0.47 ± 0.11
OATP1C1/ Slco1c1	0.14 ± 0.038	0.14 ± 0.045	1.0	0.9	0.13 ± 0.040	0.11 ± 0.080	0.85		0.7		0.27 ± 0.030	0.26 ± 0.040
OCT1/ Slc22a1	< 0.15 (ULQ)	< 0.15 (ULQ)			< 0.15 (ULQ)	< 0.15 (ULQ)					0.58 ± 0.11	0.44 ± 0.090
OAT3/ Slc22a8	0.31 ± 0.17	0.47 ± 0.28	1.5	0.2	0.086 ± 0.053	0.089 ± 0.038	1.1		0.9		0.24 ± 0.030	0.24 ± 0.010
FATP1/ Slc27a1	0.13 ± 0.068	0.086 ± 0.043	0.66	0.2	0.051 ± 0.038	0.22 ± 0.13	4.3		0.01		NQ	NQ
ENT1/ Slc29a1	0.19 ± 0.10	0.18 ± 0.13	0.95	0.8	0.027 ± 0.018	0.020 ± 0.012	0.74		0.5		0.22 ± 0.090	0.24 ± 0.040
Endothelial marker, luminal membrane												
γ-Gtp	2.9 ± 1.2	2.5 ± 0.86	0.86	0.5	0.61 ± 0.12	0.71 ± 0.11	1.1	0.2		NQ		NQ
Plasma membrane marker, abluminal												
Na^+^/K^+^-ATPase	29.0 ± 11.0	26.0 ± 2.8	0.89	0.6	15.0 ± 6.8	19.0 ± 4.1	1.3		0.2		17.0 ± 16.0	22.0 ± 28.0

Statistical significance of differences in transporter protein expression between groups was analyzed using unpaired *t*-test

*NQ*, not quantified referring to not investigated in the study; *ULQ*, under limit of quantification

^a^Abcb1 refers to both Abcb1a and Abcb1b

^b^OATP1A4 is an orthologue of human OATP1A2

The present study did not reveal any significant changes in absolute protein expression levels of the investigated ABC transporters, such as Abcb1, Abcg2, Abcc1, Abcc4, and Abca1 (Table 1). Among SLC transporters, the mean protein expression of alanine/serine/cysteine/threonine transporter (ASCT1) was more than twofold higher in the isolated brain microvessels of 5xFAD mice compared to WT animals (*p* = 0.01). In addition, the mean protein expression of 4F2 cell-surface antigen heavy chain (4F2hc) in isolated brain microvessels was 1.4-fold higher in 5xFAD mice compared to WT animals (*p* = 0.05). The protein expression of large neutral amino acids transporter small subunit 1 (LAT1) in the isolated brain microvessels of 5xFAD mice was significantly lower than that in WT animals (*p* = 0.008) (Table 1). For other SLC transporters, i.e., facilitated glucose transporter member 1 (GLUT1), long-chain fatty acid transport protein 1 (FATP1), reduced folate transporter (RFC), monocarboxylate transporter 1 (MCT1), organic anion-transporting polypeptides OATP1A4 and OATP1C1, organic anion transporter 3 (OAT3), and equilibrative nucleoside transporter 1 (ENT1), no significant differences in the mean protein expression levels between the 5xFAD and WT mice were observed (Table 1). Protein expressions of high affinity cationic amino acid transporter 1 (CAT-1) and organic cation transporter 1 (OCT1) were below the limit of quantification (Table 1).

Similar to the finding in the isolated brain microvessels, mean protein expression of ASCT1 in the crude membrane fraction of the brain prefrontal cortical tissue of 5xFAD mice was 1.9-fold higher compared to WT animals (*p* = 0.01). The mean protein expression levels of other SLC transporters, i.e., GLUT1, 4F2hc, LAT1, and FATP1, as well as all investigated ABC transporters, did not differ between the study groups (Table 2). Protein expressions of CAT-1, MCT1, OCT1, OAT3, RFC, OATP1A4, OATP1C1, and ENT1 were below the limit of the quantification (Table 2).

**Table 2:** Protein expression of SLC and ABC transporters (fmol/μg crude membrane total protein) in crude membrane fraction of the brain cortices of female wild-type (WT) mice (*n* = 14) and 5xFAD mice (*n *= 12), as well as the comparison to the protein expression levels in crude membrane of the brain cortices in female WT rats (*n* = 5) versus TgF344-AD rats (*n* = 8) [28] and female transgenic APdE9 mice (*n* = 4) versus age-matched WT (*n *= 5) mice [29]

Protein name/ gene name	Mouse brain cortical tissue analysed in the present study				Rat brain cortical tissue [28]				Mouse brain cortical tissue	
	WT mice Mean ± SD	5xFAD mice Mean ± SD	Ratio 5xFAD to WT	*p*-value	WT rats Mean ± SD	TgF344-AD rats Mean ± SD	Ratio TgF344-AD to WT	*p*-value	WT mice	APdE9 mice
ABC transporters										
Abcb1/Abcb1^a^	0.13 ± 0.061	0.15 ± 0.052	1.1	0.7	0.19 ± 0.059	0.17 ± 0.063	0.89	0.5	0.49 ± 0.19	0.31 ± 0.10
Abcg2/Abcg2	0.46 ± 0.21	0.51 ± 0.24	1.1	0.7	0.038 ± 0.028	0.039 ± 0.019	1.1	0.9	0.40 ± 0.12	0.32 ± 0.070
Abcc1/Abcc1	0.16 ± 0.033	0.16 ± 0.044	1.0	0.8	0.071 ± 0.024	0.062 ± 0.018	0.87	0.5	0.27 ± 0.070	0.24 ± 0.11
Abcc4/Abcc4	0.019 ± 0.011	0.026 ± 0.011	1.4	0.1	< 0.010 (ULQ)	< 0.010 (ULQ)			0.020 ± 0.010	0.030 ± 0.010
Abca1/ Abca1	0.12 ± 0.077	0.18 ± 0.13	1.5	0.3	0.031 ± 0.012	0.028 ± 0.0081	0.90	0.3	NQ	NQ
SLC transporters										
ASCT1/Slc1a4	3.1 ± 1.6	5.9 ± 2.5	1.9	0.01	0.12 ± 0.0029	0.075 ± 0.031	0.62	0.9	NQ	NQ
GLUT1/Slc2a1	3.5 ± 1.6	3.8 ± 1.3	1.1	0.62	2.6 ± 0.14	1.8 ± 0.35	0.69	0.8	3.7 ± 1.1	3.1 ± 1.1
4F2hc/Slc3a2	0.81 ± 0.31	0.96 ± 0.38	1.2	0.4	0.53 ± 0.13	0.33 ± 0.13	0.62	0.5	NQ	NQ
CAT-1/Slc7a1	< 0.15 (ULQ)	< 0.15 (ULQ)			< 0.15 (ULQ)	< 0.15 (ULQ)			NQ	NQ
LAT1/Slc7a5	0.73 ± 0.26	0.61 ± 0.18	0.84	0.3	0.065 ± 0.023	0.053 ± 0.021	0.82	0.3	0.25 ± 0.070	0.26 ± 0.10
MCT1/Slc16a1	< 0.10 (ULQ)	< 0.10 (ULQ)			< 0.10 (ULQ)	< 0.10 (ULQ)			NQ	NQ
RFC/ Slc19a1	< 0.15 (ULQ)	< 0.15 (ULQ)			< 0.15 (ULQ)	< 0.15 (ULQ)			NQ	NQ
OATP1A4/ Slco1a4^b^	< 0.015 (ULQ)	< 0.015 (ULQ)			0.16 ± 0.14	0.098 ± 0.069	0.61	0.5	NQ	NQ
OATP1C1/ Slco1c1	< 0.015 (ULQ)	< 0.015 (ULQ)			0.072 ± 0.035	0.041 ± 0.037	0.57	0.1	NQ	NQ
OCT1/ Slc22a1	< 0.15 (ULQ)	< 0.15 (ULQ)			< 0.15 (ULQ)	< 0.15 (ULQ)			NQ	NQ
OAT3/ Slc22a8	< 0.15 (ULQ)	< 0.15 (ULQ)			< 0.15 (ULQ)	< 0.15 (ULQ)			NQ	NQ
FATP1/ Slc27a1	0.66 ± 0.39	0.83 ± 0.41	1.3	0.4	0.74 ± 0.21	0.76 ± 0.33	1.1	0.9	NQ	NQ
ENT1/ Slc29a1	< 0.03 (ULQ)	< 0.0 (ULQ)			< 0.010 (ULQ)	< 0.010 (ULQ)			NQ	NQ
Plasma membrane marker										
Na^+^/K^+^-ATPase	72.0 ± 11.0	72.0 ± 16.0	1.0	0.9	46.0 ± 10.1	61.0 ± 14.0	1.3	0.06	170 ± 33.0	150 ± 42.0

Statistical significance of differences in transporter protein expression between groups was analyzed using unpaired *t*-test

*NQ*, not quantified referring to not investigated in the study; *ULQ*, under limit of quantification

^a^Abcb1 refers to both Abcb1a and Abcb1b

^b^OATP1A4 is an orthologue of human OATP1A2

### Comparison of Alterations in Transporter Protein Expression in the Isolated Brain Capillaries of 5xFAD Mice Versus AD Patients and Across the AD Animal Models

**Fig. 2:**
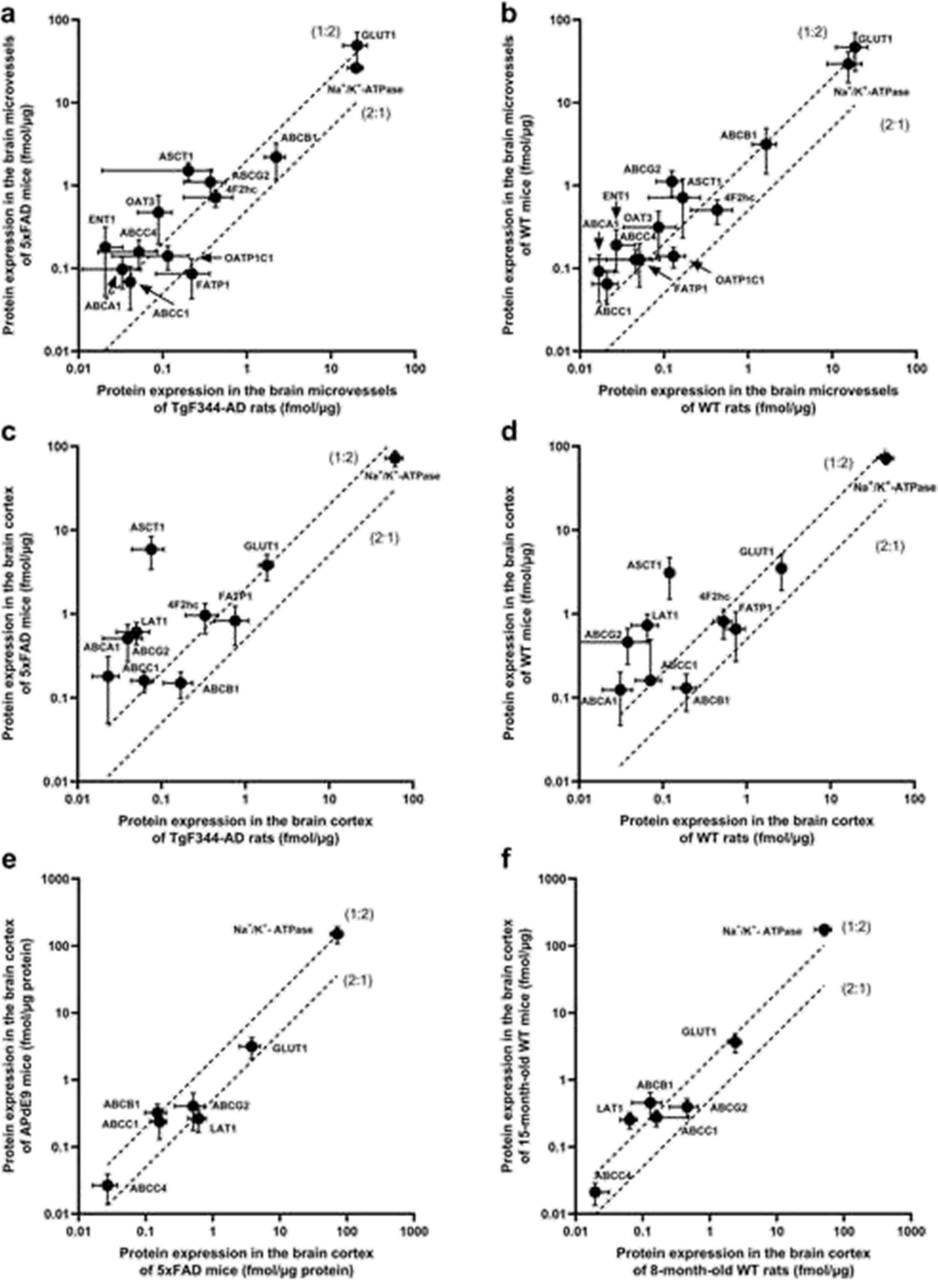
Comparison of absolute protein expression levels of the transporters in the crude membrane fraction of **a** isolated brain microvessels of TgF344-AD rats (*n* = 6) [28] versus 5xFAD mice from the present study (*n* = 6); **b** isolated brain microvessels of WT rats (*n* = 6) [28] versus 8-month-old WT mice from the present study (*n* = 6); **c** the brain cortices of TgF344-AD rats (*n *= 8) [28] versus 5xFAD mice from the present study (*n* = 12); **d** the brain cortices of WT rats (*n* = 5) [28] versus 8-month-old WT mice from the present study (*n* = 14); **e** the brain cortices of female APdE9 mice (*n* = 4) [29] versus female 5xFAD mice from the present study (*n* = 12); **f** the brain cortices of female 8-month-old WT mice (*n* = 5) from the present study versus female 16–17-month-old WT mice (*n* = 14) [29]. The top and bottom dashed lines represent a twofold higher or lower protein expression, respectively, between the studied groups. Data are expressed as mean ± SD

In the present study, we compared the changes in absolute protein expression of transporters in the isolated brain microvessels and brain prefrontal cortices of female 8-month-old 5xFAD mice and corresponding WT control mice to those previously reported in AD patients [17] and other AD animal models [28, 29] (Tables 1 and 2; Fig. 2).

The comparison of changes in protein expression of transporters in the isolated brain microvessels between 5xFAD mice and TgF344-AD rats versus corresponding WT control animals demonstrated TgF344-AD-rat-specific upregulation of Abcg2, Abcc1, and FATP1 expression and 5xFAD-mouse-specific upregulation of ASCT1 and 4F2hc as well as LAT1 downregulation (Table 1). In general, the absolute protein expressions of several transporters, i.e., Abcg2, Abcc4, Abca1, ASCT1, GLUT1, ENT1, and OAT3, were more than twofold higher in 5xFAD mice compared to TgF344-AD rat model (Fig. 2a). Similar observations were found while comparing corresponding WT rat and WT mouse transporter expression in the isolated brain microvessels of the mentioned models (Fig. 2b), indicating rather interspecies differences in the protein expression of these transporters, than AD-model-specific changes. Interestingly, FATP1 protein expression in the isolated microvessels was more than twice higher in TgF344-AD rat model compared to 5xFAD mice, while in corresponding WT rats, the expression of the protein was twice lower than in WT mice (Fig. 2a,b). These results confirm evidence for AD rat model-specific alterations in FATP1. The protein expression of some investigated transporters was under detection limits in mouse models (CAT1) and rat models (LAT1, RFC, MCT1, OATP1A4) limiting the comparison of the differences in expression of these transporters.

**Fig. 3:**
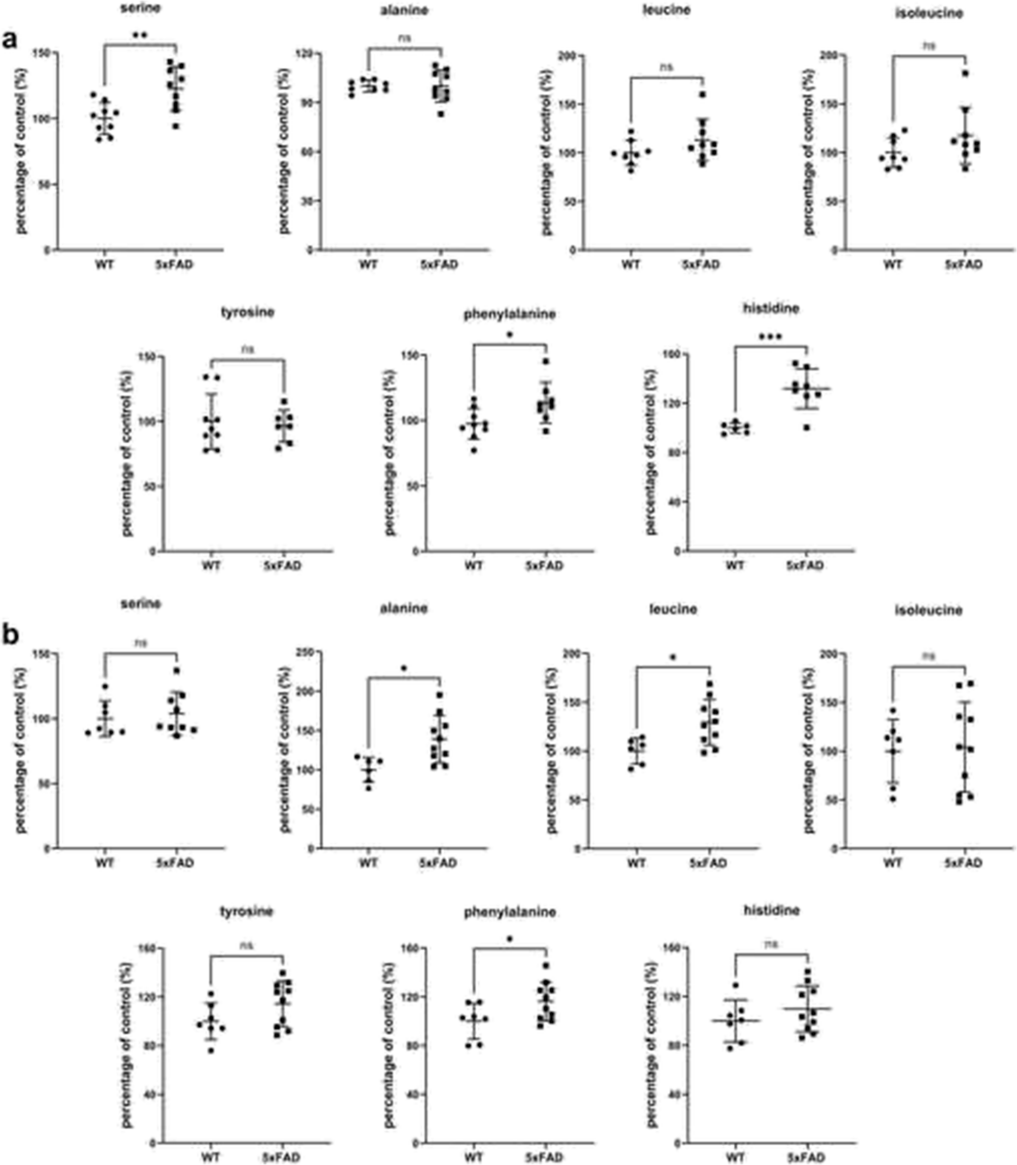
**a** Relative comparison of amino acid concentrations in the brain prefrontal cortices of 5xFAD mice (*n* = 9) and WT controls (*n* = 9). **b** Relative comparison of amino acid concentrations in the plasma of 5xFAD mice (*n* = 9) and WT controls (*n* = 9). The data are expressed as mean ± SD. Asterisks denote a statistically significant difference from the respective control (* < 0.05, ** < 0.01, ***p < 0.005, unpaired *t*-test)

For evaluation of model-specific transporter protein expression alterations in the brain cortical tissue, we compared the changes in 5xFAD mice, TgF344-AD rats expressing mutant human APP_sw_ and PS1ΔE9 genes [49], and APdE9 mice overexpressing the human APP_sw_ and PS1ΔE9 mutations [50] versus corresponding age-matched WT controls (Table 2). It should be pointed out that the comparison was done only for female animals, as the transporter expressions in mouse AD models have been reported for females, and previously, we have demonstrated sex-specific alterations in protein expression in TgF344-AD rats [28]. The comparison of changes in protein expression revealed ASCT1 upregulation specific for 5xFAD mice (Table 2), which was not observed in TgF344-AD rats or studied in APdE9 mice. Similar to the expression in brain microvessels, the absolute protein expression of some transporters such as Abcg2, Abcc1, Abca1, ASCT1, GLUT1, LAT1, and 4F2hc was more than twice higher in 5xFAD mice compared to TgF344-AD rat model (Fig. 3c). Among the mentioned transporters, the expression of ASCT1, LAT1, Abcg2, Abcc1, and Abca1 in the brain prefrontal cortical tissues of corresponding 8-month WT mice was more than double the protein expression levels in WT rats (Fig. 2d). These findings suggest inter-species differences in protein expression of ASCT1, LAT1, Abcg2, Abcc1, and Abca1 transporters and model-specific differences in GLUT1 and 4F2hc. The protein expression of some investigated transporters was under detection limits in mouse models (OATP1A4, OATP1C1) and rat models (Abcc4) limiting the comparison of the differences in expression of these transporters.

The comparison of absolute protein expression levels of four ABC and two SLC transporters in the brain cortical tissue of 8-month-old 5xFAD to another transgenic AD model, 16–17-month-old APdE9 mice, revealed only twice higher protein expression of Abcb1 in APdE9 mice compared to 5xFAD mice (Fig. 2e). The same difference in expression of the transporter was observed while comparing the corresponding WT control groups indicating age-dependent changes in Abcb1 protein expression (Fig. 2f).

The direct comparison of absolute protein expression of the transporters in the isolated brain microvessels of animal models to AD patients was not possible, as the methods used for membrane isolation in these studies were different. Thus, we compared the changes in protein expression levels in the isolated brain microvessels between mice (5xFAD mice vs. WT controls) and humans (sporadic AD patients vs. non-demented individuals) [17]. Thus, LAT1 downregulation in the isolated brain microvessels of 5xFAD mice was not observed in AD patients. The expression of upregulated ASCT1 and 4F2hc transporters in 5xFAD mice was not investigated in AD patients. The downregulation of the protein expression of MCT1 in the isolated brain microvessels of AD patients was not confirmed in 5xFAD mice.

### Changes in Amino Acid Levels in the Brain and Plasma of 5xFAD Mice

As altered protein expressions of ASCT1 and LAT1 in the isolated brain microvessels of 5xFAD mice vs. WT controls were revealed in the present study, we measured brain prefrontal cortical and plasmatic concentrations of the substrates of these transporters in both study groups. The following amino acids were measured and compared between 5xFAD mice and WT controls: serine and alanine, as substrates of ASCT1 [51, 52]; and leucine, isoleucine, tyrosine, phenylalanine, and histidine, as substrates of LAT1 [49]. As shown in Fig. 3a, serine brain concentrations were more than 20% higher in 5xFAD brains than in WT controls (*p* = 0.005). In addition, brain prefrontal cortical levels of phenylalanine and histidine were 13% (*p* = 0.003) and 30% (*p* = 0.0005) higher, respectively, in 5xFAD mice compared to WT animals. The concentrations of other amino acids in the brain prefrontal cortical tissue did not differ significantly between the study groups (Fig. 3a).

In the plasma, significantly increased levels of alanine (by 39%, *p* = 0.01), leucine (by 30%, *p* = 0.01), and phenylalanine (by 16%, *p* = 0.05) were observed in 5xFAD mice compared to WT controls (Fig. 3b). The concentrations of serine, histidine, isoleucine, and tyrosine did not demonstrate significant differences in the plasma of 5xFAD mice vs. WT controls (Fig. 3b).

## Discussion

In the present study, we provided quantitative information about absolute protein expression of ABC and SLC transporters in isolated brain microvessels and brain prefrontal cortical tissues of 5xFAD mice and age-matched WT animals, and compared the data to previously reported changes in AD patients and animal models. The study possesses several important features such as (1) the transporter expression characterization of one of the most commonly used familial AD mouse model, 5xFAD, reproducing Aβ pathology and inflammation as confirmed in the present study; (2) investigation of the transporter expression in both the isolated brain microvessels and the brain prefrontal cortices representing brain parenchyma in the same animals; (3) investigation of changes in the brain and plasmatic levels of the transporter substrates, for which altered protein expression was observed, in the same animals; and (4) providing information on quantitative absolute protein expression of the transporters using the state-of-the-art highly selective, sensitive, and reproducible LC–MS/MS-based QTAP approach [30].

The study revealed significant alterations in protein expression of amino acid transporters at the NVU cells of 5xFAD mice compared to WT controls. Thus, twice higher expression of alanine/serine/cysteine/threonine transporter 1 (ASCT1, encoded by Slc1a4), a Na^+^-dependent neutral amino acid transporter [53, 54], was found in both isolated brain microvessels and brain cortical tissue of 5xFAD mice compared to WT controls. Although ASCT1 protein expression was reported in the isolated human brain microvessels [55], capillary endothelial cells of embryonic and neonatal mouse brains [52], and rodent astrocytes and neurons [51, 56, 57], alterations in ASCT1 expression and function in the NVU cells in AD patients have not been investigated. In our previous study in the rat model of AD, TgF344-AD rats, we had not observed any changes in transporter expression in the isolated brain microvessels and brain cortices as compared to age-matched WT controls [28] indicating that changes in ASCT1 protein expression in the NVU are model-specific.

ASCT1 is responsible for the efflux of L-serine and other neutral amino acids from astrocytes [51, 52]. Consequently, L-serine is transported into neurons via a currently unidentified transporter(s), and metabolized to D-serine by serine racemase [58]. ASCT1 mediates efflux of D-serine, a high affinity substrate, from neurons to extracellular space [59, 60], where D-serine binds to *N*-methyl-D-aspartate (NMDA) receptors playing a key role in neurodevelopment, synaptic plasticity, learning, and memory [61]. The activation of NMDA receptors in AD has been shown to be implicated in leading to synaptic dysfunction [62]. D-serine levels were elevated in the brain cortex, hippocampus, and cerebrospinal fluid of probable AD patients compared to non-cognitively impaired subjects as well as APP/PS1 transgenic mice [63]. Interestingly, in the present study, along with upregulation of ASCT1 in the brain microvessels and brain prefrontal cortical tissue, we observed significantly higher levels of serine in the brain, but not in plasma, providing evidence of association of enhanced serine brain levels due to ASCT1 transporter upregulation. However, as in this study, we could not differentiate between L- and D-serine; further mechanistic research is required to confirm this hypothesis. The brain levels of another ASCT1 substrate, alanine, were the same in 5xFAD mice and WT controls. These findings can be explained by the fact that alanine is known to be a substrate of multiple amino acid transporters [64], such as B^0^AT2 (encoded by SLC6A15) and LAT2 (encoded by SLC7A8) mediating its uptake to neurons and astrocytes [65]. Overall, these findings provide the first evidence for the involvement of ASCT1 in AD pathogenesis and open new horizons for investigation of perturbations in ASCT1-related pathways in AD.

The protein expression of large neutral amino acids transporter small subunit 1 (LAT1, encoded by Slc7a5) was significantly decreased, while the expression of heavy chain subunit 4F2hc (encoded by Slc3a2) was elevated in the isolated brain microvessels of 5xFAD mice compared to WT controls. The light chain subunit (LAT1, SLC7A5/Slc7a5) is a functional subunit, which mediates Na^+^- and pH-independent exchange of large branched-chain and aromatic neutral amino acids such as leucine, isoleucine, phenylalanine, tyrosine, histidine in antiport with histidine and tyrosine [66, 67]. It is covalently linked to a heavy chain subunit (4F2hc, SLC3A2/Slc3a2), a glycoprotein that acts as a molecular chaperone localizing LAT1 and other amino acid transporters at the plasma membrane [68–70]. Moreover, 4F2hc plays a role in the processes of cell survival and integrin activation. In a QTAP study investigating the absolute protein expression of LAT1 in isolated brain capillaries in sporadic AD patients and non-demented subjects, no differences in LAT1 expression were found between the groups [17]. LAT1 protein expression and function did not differ in WT astrocytes with and without lipopolysaccharide (LPS) treatment as well as in transgenic APP/PS1 astrocytes treated with LPS [44]. Similar to the findings of this study, no changes in expression of LAT1 in the brain cortical tissue of APdE9 mice compared to age-matched WT controls were observed [29]. As LAT1 is responsible for the transport of amino acids across the BBB and within the brain, the changes in the transporter expression and functionality can affect amino acid homeostasis. In 5xFAD mice, despite a decreased expression of LAT1 in the isolated brain microvessels, we observed increased brain levels of phenylalanine and histidine as compared to WT controls. In AD patients, elevated phenylalanine brain levels but diminished histidine brain levels were previously reported [71, 72]. The increase in brain phenylalanine concentration could be explained by elevated plasma concentrations of the amino acid in 5xFAD mice. However, overall, the results of amino acid analysis do not provide direct evidence of changes in LAT1 function in the isolated brain microvessels of 5xFAD mice. As LAT1 is an extensively used transporter for the development of brain drug delivery strategies [73], future studies should elucidate whether decreased expression of LAT1 in the isolated brain microvessels of 5xFAD mice reflects the transporter functionality. The changes in LAT1 functionality might result in decreased drug delivery to the brain via LAT1 and lower drug exposure while testing new drug candidates targeting LAT1 in 5xFAD mice.

The changes in 4F2hc expression and function in AD patients have not been investigated. In our previous report, no changes in 4F2hc protein expression in the isolated brain microvessels of TgF344-AD rats were found when compared to WT controls [28]. In 5xFAD mice, we observed increased protein expression of 4F2hc in the isolated brain microvessels compared to WT mice indicating model-specific changes in 4F2hc protein expression in the brain microvessels. Interestingly, in the brain cortex of TgF344-AD rats, 4F2hc protein expression was upregulated in male animals compared to WT controls, but not in female rats [28]. In the present study, we investigated changes in transporter protein expression only in female mice, for which no differences in 4F2hc protein expression in the brain prefrontal cortex of 5xFAD and WT animals were observed, which is consistent with the findings in TgF344-AD female rats. However, it is important to further investigate sex-specific changes in 4F2hc expression and function in AD.

Interestingly, we have not observed changes in protein expression of other investigated SLC transporters as well as ABC transporters in the NVU cells of 5xFAD mice, while altered expression of FATP1, Abcg2, and Abcc1 in the isolated brain microvessels of TgF344-AD rats compared to WT controls was reported in our previous study [28]. Although both TgF344-AD rat and 5xFAD mouse models were designed to reproduce Aβ pathology, the models have different phenotypic characteristics. For example, TgF344-AD rats are characterized by the development of cerebral amyloid angiopathy, which might cause additional alterations in transporter expression in the NVU cells [28]. Moreover, in 5xFAD mice, no changes in MCT1 protein expression in the NVU cells were revealed, while significantly lower expression of the transporter, which mediates lactate trafficking among neural cells, was reported in the isolated brain capillaries from AD patients compared to non-demented subjects [17]. These findings demonstrate model-specific changes in the expression of transporters in the NVU cells and highlight the importance of selection of appropriate AD animal models, which represents the phenotype fulfilling the aims of a study as a crucial part of AD drug development.

Overall, we found model-specific changes in protein expression of amino acid transporters in isolated brain microvessels. Moreover, changes in transporter protein expression in the isolated brain microvessels observed in our study did not always represent the changes reported in sporadic AD patients [17]. These discrepancies can be explained by the fact that 5xFAD mice reproduce a simplified AD phenotype based on AD-related histopathological lesions and the utilization of FAD-associated genetic mutations. Moreover, as transporter protein expression changes can be dependent on AD stage and brain region, which were not reported for AD patients [17], one should consider the comparison to human performed in our study with caution. Therefore, future studies should address investigation of expression and function of transporters in patients at different stages of AD in order to identify which particular animal model represents such changes, in order to provide better translation of preclinical data to humans.

Some additional limitations and future perspectives should be discussed. First of all, the study focused on the investigation of changes in transporter protein expression in female mice, while we previously demonstrated evidence for sex-specific changes in transporter protein expression in the AD rat model [28]. Thus, future studies should address investigating sex-specific alterations while characterizing AD animal models. In addition, although the isolated brain microvessels mainly consist of the brain endothelial cells, some fractions of the NVU cells such as pericytes can also be present. Therefore, the changes observed in the brain microvessels do not fully represent the BBB, but a combination of the NVU cells. Moreover, as the total fraction of the brain capillary endothelial cells in the brain is negligible (0.1%) [74], we assumed that the changes in transporter protein expression observed in the brain cortical tissues represent the alterations in the combined populations of the brain parenchymal cells. However, further studies are required to investigate transporter expression and functional changes in individual brain parenchymal cell populations. Moreover, in our study, we could not differentiate between L- and D-isomers of amino acids, which makes it difficult to draw a conclusion about the perturbations in specific pathways involving the transporters. Finally, although we observed alterations in expression of ASCT1, LAT1, and 4F2hc in the isolated brain microvessels of 5xFAD mice, future studies should focus on determination of the changes in functionality of these transporters using in situ brain perfusion or cerebral microdialysis techniques as well as identification of specific molecular mechanisms underlying these changes.

In conclusion, we quantified absolute protein expression of ABC and SLC transporters in the isolated brain microvessels and the brain prefrontal cortices of 5xFAD mouse model and age-matched WT controls using LC–MS/MS-based QTAP approach. Here, we revealed significant upregulation of ASCT1 transporter in both isolated brain microvessels and brain prefrontal cortices of 5xFAD mice. These findings, along with the elevated brain levels of ASCT1 substrate, serine, provide evidence of the involvement of this amino acid transporter in AD pathogenesis. Importantly, we found altered protein expressions of other amino acid transporters such as LAT1 and 4F2hc in the isolated brain microvessels of 5xFAD mice, as well as changes in the brain levels of LAT1 substrates, phenylalanine and histidine. As LAT1 is extensively investigated as a target for drug delivery to the brain, the present study provides important information for optimal use of the 5xFAD model while testing new drug delivery strategies. All in all, the study gives insights not only into the potential role of the transporters in molecular mechanisms underlying AD, but into the optimal use of the 5xFAD mouse model during AD drug development and investigation of drug delivery strategies in AD.

## Supplementary Information

Below is the link to the electronic supplementary material.

Supplementary file1 (DOCX 1499 KB)

## Data Availability

The datasets generated during and/or analysed during the current study are available from the corresponding author on reasonable request.
