# Supplementary material for: Protein Expression of Amino Acid Transporters Is Altered in Isolated Cerebral Microvessels of 5xFAD Mouse Model of Alzheimer’s Disease
Source: Mol Neurobiol. 2022 Nov 11;60(2):732–48. doi: 10.1007/s12035-022-03111-y (PMC9849299; doi:10.1007/s12035-022-03111-y)
Supplement: Supplementary file 1 — Supplementary file1 (DOCX 1499 KB) [file 12035_2022_3111_MOESM1_ESM.docx]

**Protein expression of amino acid transporters is altered in isolated cerebral microvessels of 5xFAD mouse model of Alzheimer’s disease**

*Elena Puris^a*^, Liudmila Saveleva^b^, Izaque de Sousa Maciel^b^, Katja M. Kanninen^b^, Seppo Auriola^c†^, Gert Fricker**^a†^*

*^a^Institute of Pharmacy and Molecular Biotechnology, Ruprecht-Karls-University, Im Neuenheimer Feld 329, 69120 Heidelberg, Germany*

*^b^A.I. Virtanen Institute for Molecular Sciences, University of Eastern Finland, P.O. Box 1627, 70211 Kuopio, Finland*

*^c^School of Pharmacy, University of Eastern Finland, P.O. Box 1627, 70211 Kuopio, Finland*

^†^The authors contributed to the work equally.

***Corresponding author:** Elena Puris, Institute of Pharmacy and Molecular Biotechnology, Ruprecht-Karls-University, Im Neuenheimer Feld 329, 69120 Heidelberg, Germany;

phone: +(358)449789164; email: [elena.puris@uni-heidelberg.de](mailto:elena.puris@uni-heidelberg.de)

ORCID ID: <https://orcid.org/0000-0002-1769-389X>

**Supplementary Information**

**Supplementary Table S1.** *Primer Pairs and Expected Bands for Genotyping for the PSEN1 transgene*

|  |  |  |  |
| --- | --- | --- | --- |
| **Primer** | **Primer Type** | **Sequence (5’ – 3’)** | **Expected Band (bp)** |
| **oIMR1644** | Transgene | AAT AGA GAA CGG CAG GAG CA | 608 |
| **oIMR1645** | Transgene | GCC ATG AGG GCA CTA ATC AT | 608 |
| **oIMR7338** | Internal positive control forward | CTA GGC CAC AGA ATT GAA AGA TCT | 324 |
| **oIMR7339** | Internal positive control reverse | GTA GGT GGA ATT TCT AGC ATC ATC C | 324 |

**Supplementary Table S2.** *Primer sequences for SYBR Green qRT-PCR*

| **Gene** | **Forward primer** | **Reverse primer** |
| --- | --- | --- |
| Gfap | CACCTACAGGAAATTGCTGGAGG | CCACGATGTTCCTCTTGAGGTG |
| Aif1 | GGCTTCAAGTTTGGACGGCAGATCCTC | CATGAGCCAAAGCAGGGATTTGCAGGG |
| Il1b | GCAACTGTTCCTGAACTCAACT | ATCTTTTGGGGTCCGTCAACT |
| Gapdh | CCATGGAGAAGGCTGGGG | CAAAGTTGTCATGGATGACC |

**Supplementary Table S3.** *Probe peptide amino acid sequences and multiple reaction monitoring transitions for the LC-MS/MS analysis of target proteins.*

| **Protein/**  **gene name** | **St/IS** | **Unique amino acid sequence** | **Retention time (min)** | **MRM transitions (m/z)** | | | | |
| --- | --- | --- | --- | --- | --- | --- | --- | --- |
|  |  |  |  | **Q1** | **Q3.1** | **Q3.2** | **Q3.3** | **Q3.4** |
| **ABC transporters** | | | | | | | | |
| **Abcb1/Abcb1^a^** | St | NTTGALTTR | 8.7 | 467.7 | 719.4 | 618.3 | 561.3 |  |
|  | IS | NTTGALTT**R*** | 8.7 | 472.7 | 729.4 | 628.3 | 517.3 |  |
| **Abcg2/Abcg2** | St | SSLLDVLAAR | 27.4 | 522.8 | 757.4 | 644.3 | 529.3 |  |
|  | IS | SSLLDVLAA**R*** | 27.4 | 527.8 | 767.4 | 654.3 | 539.3 |  |
| **Abcc1/Abcc1** | St | TPSGNLVNR | 9.9 | 479.2 | 759.4 | 672.3 | 501.3 |  |
|  | IS | TPSGNLVN**R*** | 9.9 | 484.2 | 769.4 | 682.3 | 511.3 |  |
| **Abcc4/Abcc4** | St | APVLFFDR | 24.8 | 482.7 | 796.4 | 697.3 | 584.2 |  |
|  | IS | APVLFFD**R*** | 24.8 | 487.7 | 806.4 | 707.3 | 594.2 |  |
| **Abca1/Abca1** | St | FVSPLSWDLVGR | 30.1 | 688.4 | 1129.6 | 1042.6 | 247.1 |  |
|  | IS | FVSPLSWDLVG**R*** | 30.1 | 693.4 | 1139.6 | 1052.6 | 247.1 |  |
| **SLC transporters** | | | | | | | | |
| **ASCT1/** **Slc1a4** | St | ETVDSFLDLLR | 32.2 | 654.3 | 978.5 | 863.5 | 776.5 |  |
|  | IS | ETVDSFLDLL**R*** | 32.2 | 659.3 | 988.5 | 873.5 | 786.5 |  |
| **GLUT1/Slc2a1** | St | TFDEIASGFR | 21.1 | 571.7 | 894.4 | 779.4 | 650.4 | 537.3 |
|  | IS | TFDEIASGF**R*** | 21.1 | 576.7 | 904.4 | 789.4 | 660.4 | 547.3 |
| **CAT-1/Slc7a1** | St | TILSPK | 11.7 | 329.7 | 557.4 | 444.4 | 331.2 |  |
|  | IS | TILSP**K*** | 11.7 | 333.7 | 565.4 | 452.4 | 339.2 |  |
| **LAT1/Slc7a5** | St | VQDAFAAAK | 12.0 | 460.7 | 693.4 | 578.3 | 507.3 |  |
|  | IS | VQDAFAAA**K*** | 12.0 | 464.8 | 701.4 | 586.3 | 515.3 |  |
| **4F2hc/Slc3a2** | St | VAGSPGWVR | 14.6 | 464.7 | 758.4 | 701.4 | 614.3 |  |
|  | IS | VAGSPGWV**R*** | 14.6 | 469.7 | 768.4 | 711.4 | 624.3 |  |
| **MCT1/Slc16a1** | St | SITVFFK | 23.5 | 421.3 | 641.4 | 441.2 | 294.2 |  |
|  | IS | SITVFF**K*** | 23.5 | 425.3 | 649.4 | 449.2 | 302.2 |  |
| **RFC/Slc19a1** | St | DSFLVR | 17.0 | 368.7 | 534.3 | 387.3 | 274.2 |  |
|  | IS | DSFLV**R*** | 17.0 | 373.7 | 544.3 | 397.3 | 284.2 |  |
| **OATP1A4/**  **Slco1a4** | St | EVATHGVR | 5.0 | 434.7 | 640.4 | 468.3 | 331.2 |  |
|  | IS | EVATHGV**R*** | 5.0 | 439.7 | 650.4 | 478.3 | 341.2 |  |
| **OATP1C1/**  **Slco1c1** | St | DFLPSLK | 22.7 | 410.2 | 557.4 | 444.3 | 263.1 |  |
|  | IS | DFLPSL**K*** | 22.7 | 414.2 | 565.4 | 452.3 | 263.1 |  |
| **OCT1/Slc22a1** | St | ENTIYLQVQTGK | 18.1 | 697.4 | 936.5 | 773.5 | 660.4 |  |
|  | IS | ENTIYLQVQTG**K*** | 18.1 | 701.4 | 944.5 | 781.5 | 668.4 |  |
| **OAT3/Slc22a8** | St | YGLSDLFR | 27.1 | 485.8 | 807.4 | 637.3 | 550.3 |  |
|  | IS | YGLSDLF**R*** | 27.1 | 490.8 | 817.4 | 647.3 | 560.3 |  |
| **FATP1/Slc27a1** | St | LLPQVDTTGTFK | 20.5 | 660.4 | 1093.6 | 996.5 | 868.44 | 769.4 |
|  | IS | LLPQVDTTGTF**K*** | 20.5 | 664.4 | 1101.6 | 1004.5 | 876.4 | 777.4 |
| **ENT1/Slc29a1** | St | ALADPTVALPAR | 19.7 | 597.8 | 939.5 | 727.4 | 527.3 |  |
|  | IS | ALADPTVALPA**R*** | 19.7 | 602.8 | 949.5 | 737.4 | 537.3 |  |
| **Plasma membrane marker, abluminal membrane** | | | | | | | | |
| **Na^+^/K^+^–ATPase** | St | AAVPDAVGK | 10.7 | 414.3 | 685.4 | 586.3 | 489.3 |  |
|  | IS | AAVPDAVG**K*** | 10.7 | 418.3 | 693.4 | 594.3 | 497.3 |  |
| **Endothelial marker, luminal membrane** | | | | | | | | |
| **γ-Gtp** | St | LFQPSIQLAR | 22.4 | 586.8 | 784.4 | 687.4 | 600.4 |  |
|  | IS | LFQPSIQLA**R*** | 22.4 | 591.8 | 794.4 | 697.4 | 610.4 |  |

^a^Abcb1 refers to both Abcb1a and Abcb1b

St – standard, IS – internal standard

Bold letter with* denotes labelled arginine (R) or lysine (K) with a stable isotope ^13^C and ^15^N

**Supplementary Table S4.** *Optimized MRM transitions, collision energy, and cell accelerator voltage of unlabeled and isotope-labeled amino acids.*

| **^*^Amino acid** | **Precursor Ion >**  **Product Ion**  **(m/z)** | **Collision Energy (V)** | **Accelerator Voltage**  **(V)** |
| --- | --- | --- | --- |
| ^13^C^15^N tyrosine | 192.2 > 98.1 | 30 | 7 |
| tyrosine | 182.2 > 136 | 9 | 7 |
| ^13^C^15^N phenylalanine | 176.2 > 129.1 | 10 | 7 |
| phenylalanine | 166.1 > 119.9 | 14 | 7 |
| ^13^C^15^N histidine | 165.2 > 118 | 14 | 7 |
| histidine | 156.1 > 109.9 | 13 | 7 |
| ^13^C^15^N lysine | 155.2 > 90.1 | 18 | 7 |
| lysine | 146.9 > 83.9 | 15 | 7 |
| ^13^C^15^N leucine | 139.2 > 92.1 | 10 | 7 |
| leucine | 132.1 > 85.8 | 10 | 7 |
| ^13^C^15^N isoleucine | 139.2 > 92.1 | 10 | 7 |
| isoleucine | 132.1 > 85.8 | 10 | 7 |
| ^13^C^15^N serine | 110 > 63.1 | 10 | 7 |
| serine | 106 > 59.9 | 9 | 7 |
| ^13^C^15^N alanine | 94 > 47.1 | 10 | 7 |
| alanine | 90 > 43.9 | 10 | 7 |

^*^All listed non-labeled amino acids represent both L-and D-isomers.


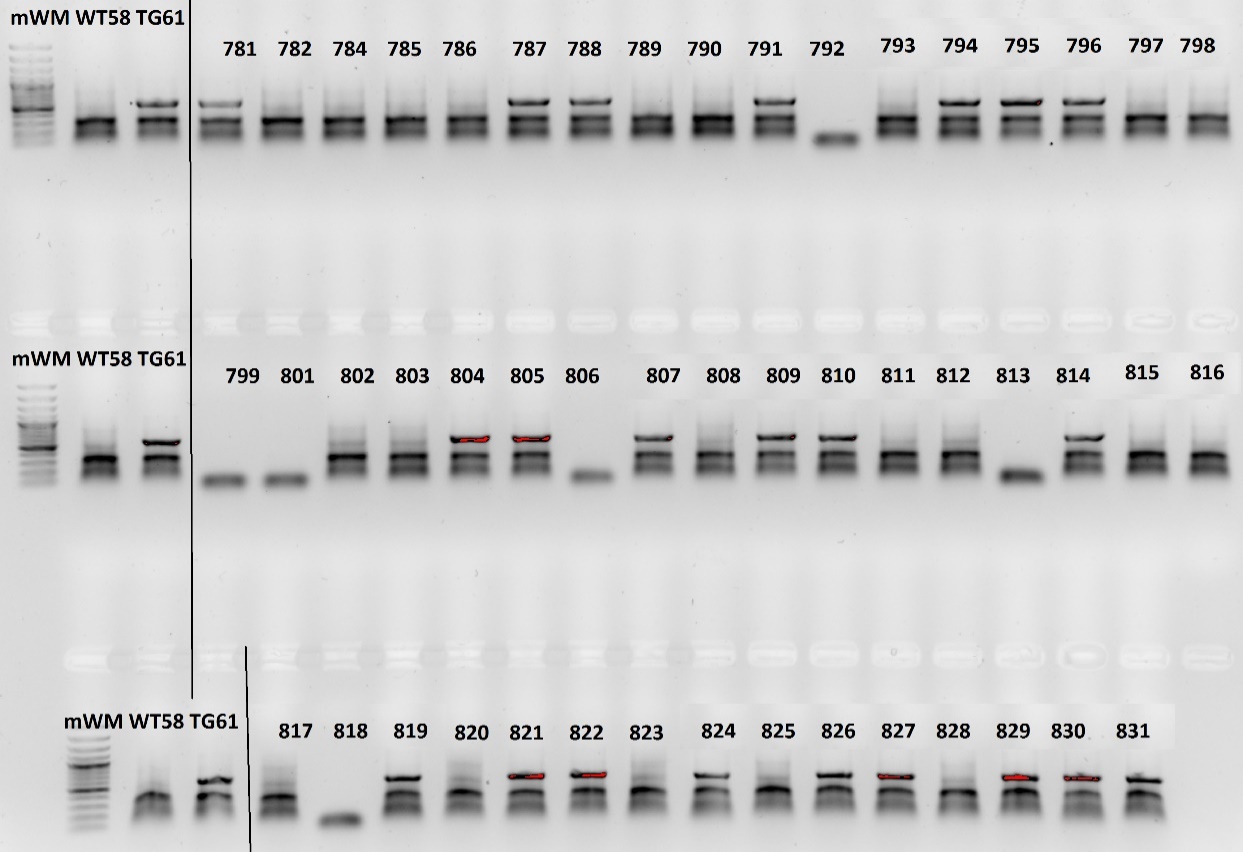


**Figure 4.** *A representative image of genotyping data. mWM - molecular weight marker (GeneRuler 100 bp Plus DNA Ladder, ThermoFisher Scientific, #SMO321), WT58-negative control, TG61-positive control, 781-798 – test samples. Transgenic samples are detected by the presence of double bands: one of the PSEN1 transgene (608 bp) and another one of the internal positive control (324 bp), while non transgenic (WT) samples lack the PSEN1 transgene band (608 bp).*


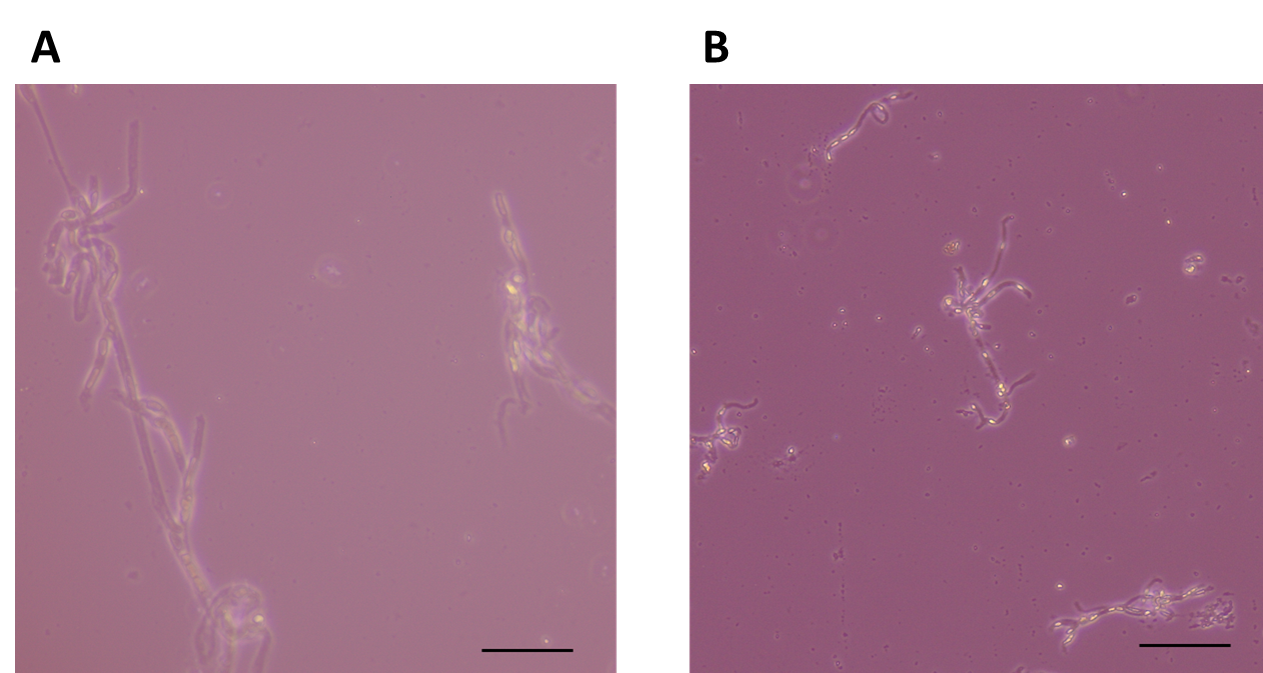


**Figure 5**. *The photographs of microvessels isolated from the fresh mouse brain cortices. (A) 20 × magnification, scale bar = 100 μm; (B) 10 × magnification, scale bar = 200 μm.*
